# Exploring experiences of psychological distress among Iranian parents in dealing with the sexual behaviors of their children with autism spectrum disorder: a qualitative study

**DOI:** 10.25122/jml-2021-0290

**Published:** 2022-01

**Authors:** Mojgan Masoudi, Raziyeh Maasoumi, Mohammad Effatpanah, Nicola Luigi Bragazzi, Ali Montazeri

**Affiliations:** 1. Department of Midwifery and Reproductive Health, School of Nursing and Midwifery, Tehran University of Medical Sciences, Tehran, Iran; 2. Nursing and Midwifery Care Research Center, School of Nursing and Midwifery, Tehran University of Medical Sciences, Tehran, Iran; 3. Pediatric Department, Tehran University of Medical Sciences, Tehran, Iran; 4. Department of Mathematics and Statistics, York University, Toronto, Canada; 5. Academic Center for Education, Culture and Research (ACECR), University of Science and Culture, Tehran, Iran

**Keywords:** sexual behavior, parents, autism, Iran, ASD – Autism spectrum disorder, HFA – High-Functioning Autism, IQ – intelligence quotient

## Abstract

Sexual behavior is influenced by social and communication deficits in autism spectrum disorder (ASD) and is a serious challenge for parents who lack effective strategies for providing sexual education to their children with ASD. The purpose of this study was to explore Iranian parents' experiences of psychological distress in dealing with the sexual behaviors of their children with ASD. This qualitative study was designed following the conventional content analysis approach. Semi-structured and in-depth interviews were conducted with 27 parents of children with ASD aged 8–34 years. All interviews were audio-recorded and transcribed verbatim. The data were collected through purposeful sampling and continued until data saturation. The worries theme was extracted from data interpretation using qualitative content analysis, and this theme entailed four subthemes: 1) sexual vulnerability, 2) unintended social consequences, 3) psychological suffering, and 4) confusion about the future of a child's sex life. This study emphasized the importance of paying attention to parents' concerns about the sexual behaviors of children with ASD. Parents' psychological distress is a major obstacle to proper coping with sexual behaviors, and using coping strategies may help reduce psychological distress in parents of children with ASD. Therefore, it is necessary to design, implement, and evaluate culture-appropriate educational programs to address parents' concerns regarding the sexual health of a child with autism.

## Introduction

Autism spectrum disorder (ASD) is a developmental disability associated with social and communication disorders, limited interests, and repetitive behavior [1]. The number of reported cases of ASD has increased over the past 30 years, and the current prevalence is estimated to be at least 1.5% in developed countries [2]. The CDC’s Autism and Developmental Disabilities Monitoring Network reported that approximately one in 54 children aged 8 years in the United States was diagnosed with ASD [3]. The parents of young people who differ from their peers in various developmental aspects (mental, physical, and social), such as those with ASD, may face unique challenges [4]. In particular, the parents of children with autism experience unparalleled challenges and face various negative psychological consequences. Although autism affects the entire family, and despite the correlation between the behavior of ASD patients and their family’s well-being, few studies have examined family dynamics in the families of people with ASD. The term High-Functioning Autism (HFA) is used for people with ASD without an intellectual disability who often have an estimated IQ of 70 or higher [5]. New research revealed this group’s unique sexual profile and sexual functioning with a similar level of interest in sex as healthy individuals [6, 7]. In societies, cultures, and religions, the concept of inappropriate sexual behavior is different. Lack of awareness of appropriate behavior or communication is one of the reasons for inappropriate behavior. People with autism have impaired awareness, social interaction, and communication skills that can lead to the display of inappropriate sexual behaviors in terms of cultural/religious norms [8].

Sexual desire and experience combined with the social deficit associated with ASD can lead to unwanted sexual contact in many ASD patients due to their impaired ability to differentiate between private and public behaviors and misunderstanding others’ intentions [9]. The literature reviews show that stress among parents of children with ASD comes from their behaviors and from being judged by society because of their children’s behaviors [10]. A systematic review by Beddows & Brooks reported a high prevalence of masturbating and indecent public exposure of genitalia and inappropriate romantic behaviors in this group, which caused problems and concerns for their families regarding sexual behavior [11]. The behavioral problems of a child with ASD can thus lead to high levels of parental stress, anxiety, and depression [12]. The systematic review and meta-analysis by York. *et al.* showed that, in general, the associations between children’s emotional and behavioral problems and parent mental health problems and parenting stress are robust, and future research should focus on further investigating the factors involved in these relationships [13].

Regarding this issue, specialists should address parents’ concerns and needs seriously. Parents’ thoughts and feelings about their children’s sexual behavior can lead to an appropriate response to these behaviors if combined with knowledge. Age-appropriate sexual behaviors are guided mainly by proper parental reassurance and education, but sexual behavior disorders often require further evaluation and protection of the child from possible abuse or neglect [14]. Parents’ communication with their children about sexual issues is an ongoing process of providing information and values about gender and sex-related issue [15]. According to literature, parents view themselves as the main individuals responsible for giving sexual education to their children with ASD but report that their access to helpful resources and support is insufficient or inadequate [16].

There are no accurate statistics on the prevalence of autism in Iran. In a national autism screening program on 1.32 million 5-year-old children in three academic years from 2006 to 2009, the prevalence of autism was 6.26 per 10,000 of the population [17]. In this country, values and cultural norms influence Iranian sexual behavior: for instance, vaginal sexual intercourse is not common sexual behavior before legal marriage. Even friendship with the opposite sex is not acceptable in Iranian families [18–21]. Furthermore, behaviors such as masturbation are forbidden/deprecated in Iranian culture, and there are many recommendations to avoid it [21, 22]. It is evident from the literature that there were insufficient findings on stress and emotional well-being of parents due to sexual behaviors of children with ASD; therefore, this study aims to address that gap. The findings of this study could help individuals better understand parents’ experiences following exposure to the sexual behaviors of children with ASD in an Iranian cultural context. Furthermore, this study could provide ideas for design and implementation of sexual education programs for Iranian parents. Therefore, this qualitative study was the first study aimed to explore the experiences of psychological distress among Iranian parents, in dealing with the sexual behaviors of their children with ASD.

## Material and Methods

### Participants

All parents participating in the present study went to educational and rehabilitation centers affiliated to the central branch of the Iranian Autism Association in Tehran and its branch in Khorramabad in Lorestan Province of Iran. Purposive sampling was performed until data saturation occurred. Twenty-seven subjects were interviewed. The inclusion criteria for parents consisted of a definitive diagnosis of High-Functioning Autism (HFA) in the child with intelligence quotient (IQ) of 70 or greater and with verbal abilities, the ASD child performing behaviors appearing as sexual, the lack of concomitant mental and physical disability or other psychiatric disorders in the child, the lack of chronic mental and physical illness in the parents, and being literate.

### Procedure

The current study was the preliminary part of a multistage mixed-methods design study that highlighted the experiences of Iranian parents in dealing with the sexual behaviors of a child with autism. Parents’ experiences of coping with the sexual behavior of their children were mapped via semi-structured interviews. Semi-structured interviews are an appropriate tool because they provide detailed information about participants’ interpretations and perceptions of their experiences by describing their daily life events [23]. Therefore, the research team decided to use individual interviews to collect data, and in this study, 27 individual interviews were conducted with participating parents. Individual interview length varied from 45 to 110 min with a mean length of 65 min, and the interviews were audio-recorded with participants’ permission. A parents’ interview guide form was designed to collect data in the present study. The interviews began with a question about coping with sexual behavior. They continued with follow-up questions such as how the parents felt about the behavior, how much knowledge the parents had about the sexual behaviors of a person with ASD, and how the parents reacted to the sexual behavior of their child with ASD. The participants were then encouraged to share their experiences in dealing with the sexual behaviors of their children through questions such as “Can you elaborate on that?” and “What do you mean?”.

### Data analysis

In this study, all data obtained from individual interviews were analyzed based on the six-step conventional content analysis according to Graneheim and Lundman, concurrently as they were collected [24]. Initially, each interview was transcribed word by word, and then the text was read several times to immerse in the data and gain an insight into them. The meaning units were then identified based on the study objectives and coded considering the manifest and latent content of the data. Finally, these codes were categorized into broader subcategories based on their similarities and differences. This data reduction and data abstraction process continued until the extraction of themes.

### Trustworthiness

The views of Lincoln and Guba were used to ensure the accuracy and reliability of the data, and the criteria of credibility, transferability, dependability, and confirmability were considered [25]. The validity of the present findings was ensured by collecting and analyzing data over a long period of 14 months. Member checks and peer reviews were also used to ensure data validity; in these steps, four parents and two of the research colleagues reviewed the data and findings and confirmed that their understanding of the data was consistent with our interpretations. In addition, a preliminary review of the text was conducted to prevent the researchers from having biases in their data collection and analysis. Finally, the rigor of the study was also verified by the accurate recording of the data collection and analysis steps to facilitate others’ monitoring of our research activities. The present study was conducted using conventional qualitative content analysis from November 2019 to August 2020. Qualitative research is as important as quantitative research, provided that it is scientifically accurate, and there are good examples of qualitative research which have significantly improved our understanding of autism [26]. Furthermore, qualitative research helps understand the human condition in the face of various phenomena and different situations [27]. At baseline, the researcher introduced herself to the interviewees, explained the study objectives, and ensured the confidentiality of participants’ names and details and the collected data.

## Results

The mean age of the parents participating in this study was 41.00±9.00 years. Most parents were married, and only one mother was divorced; also, most parents had academic education (54%), were female (85%), and were housewives (59%). The mean age of children with ASD was 14.00±6.00 years. They were mostly male (75%). Moreover, most parents (n=62%) had two children. All parents participating in this study were Iranian and Muslim. 18.5% of participating parents (n=5) had less than $4.00, 66.7% (n=18) had $4.00 to $6.00, and 14.8% (n=4) had more than $6.00 in monthly household income. Participant demographics are provided in Table 1. Based on the present findings, one central theme was found as “worries”. This theme entailed four subthemes, including “sexual vulnerability”, “unintended social consequences”, “psychological suffering”, and “confusion about the future of a child’s sex life” (Table 2).

**Table 1.** Demographic details of the parents participating in the interviews.

**Participant number**	**Parent**	**Age (year)**	**Education**	**Employment status**	**Number of children**	**Age of the child (year)**	**Gender of the child**
(1)	Mother	41	High school diploma	Housewife	2	7	Male
(2)	Mother	42	High school diploma	Housewife	2	21	Male
(3)	Mother	32	High school diploma	Housewife	1	11	Male
(4)	Mother	42	High school diploma	Housewife	3	15	Male
(5)	Mother	47	Bachelor’s degree	Employed	1	14	Female
(6)	Mother	50	Bachelor’s degree	Employed	2	24	Male
(7)	Mother	44	High school diploma	Housewife	2	25	Male
(8)	Mother	36	High school	Housewife	2	15	Male
(9)	Mother	57	Master’s degree	Employed	2	15	Male
(10)	Father	41	PhD	Employed	2	16	Male
(11)	Mother	49	High school diploma	Employed	3	17	Male
(12)	Father	37	Bachelor’s degree	Self-employed	2	10	Female
(13)	Mother	36	Bachelor’s degree	Housewife	2	7	Male
(14)	Mother	38	High school	Housewife	1	8	Male
(15)	Father	74	Bachelor’s degree	Retired	1	34	Male
(16)	Mother	38	Bachelor’s degree	Housewife	1	14	Male
(17)	Mother	36	Bachelor’s degree	Housewife	2	12	Female
(18)	Mother	50	PhD	Employed	2	15	Female
(19)	Mother	33	Bachelor’s degree	Housewife	2	8	Male
(20)	Mother	40	High school	Housewife	2 autistic children	10, 17	Male
(21)	Mother	42	High school	Housewife	2	13	Male
(22)	Mother	50	High school diploma	Housewife	1	16	Male
(23)	Mother	31	Bachelor’s degree	Self-employed	2	12	Female
(24)	Mother	28	Bachelor’s degree	Housewife	1	9	Male
(25)	Father	45	Bachelor’s degree	Employed	1	18	Male
(26)	Mother	28	High school diploma	Housewife	2	7	Female
(27)	Father	39	Bachelor’s degree	Employed	2	14	Male

**Table 2.** The main theme and sub-themes, and extracted codes.

**Theme**	**Subtheme**	**Extracted codes**
**Worries**	**Sexual vulnerability**	Fear of child’s inability to understand the risks and social threats
		Concern about sexual behaviors to provoke their child
		Endangering the child to a silent victim of sexual abuse
	**Unintended social consequences**	Anxiety about improper reaction of others to behavior
		Fears of family social isolation
		Concern about the bad judgment of family upbringing
	**Psychological suffering**	Stressful and anxious reactions to behavior
		Feeling unable to control the sexual behavior and helpless
		Upset of loss of parental marital emotional relationships
	**Confusion about the future of child's sex life**	Fear of Cultural challenges
		Concern about lack of commitment to romantic relationships
		Disappointment with the child's ability to understand and satisfy the sexual needs of others

### Theme Worries

#### Sexual vulnerability

The finding of the present study showed that all parents considered people with ASD to be at greater risk of sexual abuse compared to typically developing children due to perceptual problems such as inability to read intentions and to understand the risks and social threats: “*His mother was saying ‘What will happen to my child eventually? He will get sick going after these things [sexual intercourse]’. Autistic children don’t act on their will; they don’t understand, they don’t distinguish good from bad; they make friends, either with boys or girls and then, they are tricked into anything and get sex*” (P-15, Father of a 34-year-old male).

Most parents feared that the autistic child would provoke behaviors due to their sexual and emotional needs as well as their inability to understand the socio-cultural norms and rules of their community to something like kissing and hugging others, which can incite others to abuse the child sexually: “*My son sometimes rubbed his penis in the presence of my nephew. My nephew noticed my son’s behavior. One day when we went on a picnic with my brother’s family, my nephew took my autistic son with him behind a tree and told him ‘Undress’ and my son obeyed. When my son took off his shorts at my nephews’ orders, I, who had been secretly following them, immediately intervened and shouted at them. My nephews ran away, but my son was scared and just said, ‘What happened, mom?’* ” (P-20, Mother of a 17-year-old male).

On the other hand, parents were concerned about their child becoming a silent victim and tempting prey for abusers due to the lack of social interaction of children with ASD and inability to understand and report sexual abuse: “*I do not leave my daughter alone with anyone for a minute; even when the earthquake struck, I did not leave her room because her leg was broken. I stayed in front of her, I do not even let her cousins, who are small, kiss her face. My daughter does not realize that she could be harmed. If someone hurts my daughter, she will not notice and complain. I do not trust even close relatives because they know that my daughter has a problem; they are more dangerous*” (P-12, Father of a 10-year-old female).

#### Unintended social consequences

Most parents stated that because most people with autism seem to be healthy, society does not accept their sexual behaviors that are contrary to the religious and cultural values of society. For example, in Islam, kissing, hugging, and touching the bodies of girls and women, except for “Maharam” men (father, brother, grandfather, cousin, husband, and son), is considered a sin and is forbidden. Therefore, the competence of parents to raise their children is questioned. By isolating the families of these people, they try to prevent the negative educational effects of these behaviors on other people in the society: “*I am very scared; when my son meets the girls that he loves, he hugs and kisses them, and the families of these girls, despite knowing that my son has autism, get very angry with his behavior. Unfortunately, my son is often insulted and even beaten because of this behavior, but they are right because their reputation is lost*” (P-14, Mother of an 8-year-old male).

According to the findings of this study, family interaction with the community was very important and valuable for most parents: “*I think a lot about my son being seen by others while masturbating because they don’t respond well to his behavior. They will reject our family, and we are constantly forced to change our place of residence*” (P-10, Father of a 16-year-old male).

According to most parents, one of the factors that could destroy the family’s position in society was the misunderstanding of their autistic child behavior: “*I’ve cut connections with many people. I’m very scared of this issue. When we’re in public, I’m always watching out for my son. There really is no proper understanding of autism and of an autistic child’s behavior in our society. They judge them as rude kids and treat them badly*” (P-19, Mother of an 8-year-old male).

In other words, one of the challenges that most parents expressed in this study was misjudging their parental competence: “*My sister-in-law’s father had gone into my daughter’s room and caught her arousing herself. She came to me and said, ‘You and her father must have done something in front of your daughter to make her do this. I said, ‘No, we haven’t! What kind of people are we to do such things in front of her?’ I got very upset because of his comment*” (P-23, Mother of a 12-year-old female).

#### Psychological suffering

In this study, most parents stated that because they are unable to accept the sexual behaviors of their children, they experience unpleasant psychological effects, such as depression, helplessness, guilt, anger, fear, and disgust, and most of the time, anxiety: “*When I find out that my son is rubbing his penis, I experience a state of stress, nausea and severe anxiety. I ask myself why has he become like this? How can he understand anything about sex?*” (P-2, Mother of a 21-year-old male).

Also, in this regard, one of the participants expressed her feelings like this: “*I love my son very much, but ever since he tried to stick his genitalia to my body, I experience the worst feeling of my life –the feeling of disgust toward my own child*” (P-4, Mother of a 15-year-old male).

Based on the present findings, most of the participating parents felt unable and helpless to control the sexual behaviors of their autistic children: “*It’s hard; I’m very anxious. My son goes into his room, crawls under a blanket, and masturbates. If his work isn’t finished, he gets angry and yells and says, ‘get out of my room, or I’ll break the windows’. And I know there is nothing I can do about it*” (P-16, Mother of a 14-year-old male).

In this study, most parents complained of decreased sexual desire and intimacy in their married life. Most of them shared a bedroom with the autistic child to care for and protect him or her because they were likely to see their parents having sex, and they blamed themselves for the sexual behavior of the child: “*I made a mistake, my son has seen a behavior from me and his mother that has been provoking in such a way that I think it is wrong to have a relationship with my wife at all, my desire for her has decreased a lot*” (Participant 27, Father of a 14-year-old male).

The increase of conflict and mistrust in couples’ relationships due to blaming each other regarding the child’s sexual behaviors has gradually reduced the parents’ sexual desire: “*When my son sticks his body to others or touches and plays with his penis, I quickly check my husband’s phone, but sometimes my husband understands and is very upset because I know my son must have seen a scene that he is imitating. I myself observe a lot, even in the type of clothes I wear, but men do not observe much. To prevent my child from being harmed, I sleep in the same room at night, and my husband sleeps in the hall next to my son*” (P-9, Mother of a 15-year-old male).

#### Confusion about the future of a child’s sex life

All parents were concerned about the future of their child’s sexual life because individual sexual behaviors such as masturbation and premarital sex are not acceptable in terms of the socio-cultural structure of Iranian society. Also, the inability of people with autism to understand the needs of their sexual partner and their lack of commitment to continue the marital relationship are important barriers to the desire of other people to have formal sex and legal marriage with them. Cultural barriers are one of the main problems in meeting the sexual needs of people with autism: “*My son says that if I do not get married, I will kill myself. They are the ones who always need care. If a healthy person wants to marry them, they may do so only to earn money or the purpose of marriage is to deceive, and even if they marry an autistic person, that is a disaster and masturbation is also an ugly habit*” (P-7, Mother of a 25-year-old male).

Another parent participating in the present study expressed concern about the sensitivity and cultural significance of girls’ virginity at the time of marriage and the prohibition of premarital sex in society: “*In our culture, if a girl loses her virginity, even if she has autism, it is very difficult for the family. I’m afraid my daughter will be raped and lose her virginity. My daughter does not understand the value of virginity*” (P-17, Mother of a 12-year-old female).

One of the most common concerns of the parents participating in this study was the special emotional and romantic status of people with autism, which is the inability to establish and maintain a deeply emotional and romantic relationship with a sexual partner or spouse: “*I do not know how my son reacts to these desires; I’m afraid that he can’t control his sexual needs. When he becomes an adult; I always think that my son might hurt himself; I cannot think of my son’s marriage because of his circumstances. Autistic people cannot love their spouse, and they cannot be committed and faithful to continue sex. If they have sex, they can easily break it off and forget about it. On the other hand, no one wants to marry such a person*” (P-13, Mother of a 7-year-old male).

Most of the parents participating in this study consider their child’s inability to understand and meet the emotional and supportive needs of their spouse or sexual partner as an obstacle to a successful marriage and the legitimate satisfaction of their child’s sexual needs: “*All women want their husbands to know what they expect from a sexual relationship. If their husband discovers and fulfills these expectations, they will enjoy this relationship. My son cannot do this marital duty at all, so no woman enjoys this one-way relationship. In the future, his wife would get frustrated and leave my son*” (P-6, Mother of a 24-year-old male).

## Discussion

The findings of the present study showed the profound worry of parents regarding the sexual behaviors of a child with ASD. These concerns alone can result in parental distress. Worry can be defined as an aversive emotional experience characterized by repeated, unpleasant thoughts concerning the future. Psychological distress is a term used to describe an emotional state characterized by psychological symptoms of mental health problems in the areas of depression and anxiety [28, 29]. Multiple processes may explain the association between a child’s behavioral difficulties and psychological distress in parents of children with ASD [30]. Based on an implicit interpretation of most parents’ statements, the root of these worries was the interaction, communication deficits, and inability to understand social rules and norms of children with ASD, allowing them to display abnormally appearing and provocative sexual behaviors. On the other hand, their inability to understand the goals and intentions of others makes sexual abuse of people with ASD easy and tempting for others. In other studies, most parents were concerned about the sexual abuse and sexual health of their child with ASD [9, 16, 31], and according to parents, the vulnerability of their children is due to their perceptual inability or weakness in judging the negative intentions of others; in other words, their inability to understand others’ intentions to prevent sexual abuse [4, 9].

In this study, another worry present among most parents was the unintended social consequences of the sexual behavior of their children. Parents found that their child’s psychological and behavioral functioning results from not understanding privacy and boundaries and having sensory problems, which affect the direction of their sexual behaviors and lead to society’s misunderstanding of these behaviors. All parents were concerned about others using physical punishment and insults to respond to their autistic child’s sexual behavior. Also, the severe reaction of others to the sexual behavior of a person with ASD is one of the influential factors in aggravating and increasing sexual behavior in these people [14, 32, 33]. They were mainly concerned about their family’s social stigma and isolation due to misunderstanding the sexual behaviors of their children and wrong judgments about parenting.

In this study, most parents were concerned about their children with ASD displaying behaviors that others could misinterpret as an invitation to sex [9]. In another study as well, most of the parents stated that they suffered from social isolation due to the community’s negative attitude toward these patients and the parent’s inability to control their child’s behavior properly [34], and for this reason, they tend to reduce social activities [35]. The present findings showed parents worry about the occurrence of psychological suffering in the family, and according to most parents, they experienced unpleasant feelings such as anxiety, fear, guilt, and helplessness following the encounter with the sexual behavior of their child. According to literature, the parents of children with ASD suffer from extremely negative psychological consequences, such as depression, anxiety, and emotional distress [14, 16, 36]. They have high levels of stress and low levels of mental health, and the behavioral problems of people with ASD are associated with parents’ stress and anxiety [12]. Most of the parents participating in the present study felt unable to control the sexual behaviors of the autistic child. Based on available studies, the presence of stress in the family increases the child’s sexual behavior unless the parents learn that their child’s sexual behavior is not caused by behavioral disorders or their subjugation to sexual abuse [14]. Researchers found that they need to promote mental health in parents of children with ASD. Sigurðardóttir *et al.* revealed the need to provide education and support to the parents of autistic people to manage their children’s behavioral problems and implement interventions to reduce their stress, anxiety, and depression [37]. Therefore, high parental stress may impair the ability to use effective strategies to establish behavioral discipline in autistic children, affecting and exacerbating these children’s abnormal behaviors [38].

One of the concerns expressed by the parents participating in this study was the impact of sexual behaviors of autistic children on further reduction of intimate marital status and parental sexual relationship. Because caring for an autistic child requires a lot of energy and time, and this care often makes parents tired and preoccupied, they do not have enough time to spend intimate marital time as well as intercourse. Our results are consistent with previous studies about the effect of an autistic child on reducing parental intimacy [34, 39, 40]. Also, most participants considered the observation of intimate relations of parents as the cause of the sexual behavior of their autistic child. Therefore, by reducing the intimate relationship and even separating their bedroom and their spouse, they tried to prevent the sexual behaviors of the affected child. On the other hand, most parents blamed each other for the sexual behaviors of an autistic child and increased the possibility of tension in the couple’s relationship and the emotional separation of the spouses. According to studies, parents may blame each other for the problems of a disabled child, and such mutual accusations can cause tensions and conflicts in family relationships and lead to parental divorce [34, 41–43].

One of the main concerns of parents in this study was confusion about the future of an autistic child’s sex life. Most parents thought about how their autistic child could meet his or her sexual needs, because according to the culture of Iranian society, sexual intercourse is possible only through formal marriage, and masturbation is not acceptable, whereas the results of studies indicate the failure of autistic people in marriage and its continuation [44–46]. Iranian society has a traditional and religious culture, and sex has cultural restrictions, prohibitions, taboos, sexual activities such as masturbation, erotic behaviors, and transgender relationships, as well as premarital dating and the losing of virginity before formal marriage are culturally forbidden [18, 22, 47]. Moreover, the Iranian culture generally deplores the idea of children as sexual beings [48]. In this study, most parents were concerned about an autistic child’s inability to commit to and be responsible for a romantic relationship because intimate interpersonal relationships require interaction, communication, and commitment; this means a broad and long-term orientation [49]. However, the characteristics of people with autism are not conducive to these relationships [44]. The inability of autistic people to understand the emotions of others often leads to their inadequate response to sexual needs and, as a result, their sexual partner’s dissatisfaction. Findings from literature show that the spouses of autistic people, despite knowing that their spouse was attractive before marriage, struggle with loneliness after marriage. Experiences of deprivation and forgetfulness and repeated struggles of autistic spouses alone have led to a major crisis in marital life [44].

The present study has a limitation that should be considered in interpreting the findings. Due to the higher prevalence of sexual behavior in high-functioning autism, this study was performed on the parents of this group of patients without dividing the parental issues by their ASD child’s age group.

Based on the findings of the present study, it is recommended that future research explores effective strategies to prevent and alleviate parents’ concerns about the sexual behaviors of autistic children.

## Conclusion

This study emphasized the importance of paying attention to parents’ concerns about the sexual behaviors of children with ASD. Parents’ psychological distress constitutes a significant obstacle to proper coping with sexual behaviors and proper control and guidance of sexual needs and behaviors of autistic children. Parents were more concerned about the reactions and social consequences of autistic child sexual behaviors, especially sexual abuse and family isolation. Therefore, health policymakers should focus on implementing educational programs for parents and different segments of society to understand ASD and sexual behaviors and prevent sexual abuse of people with ASD.

## Acknowledgments

### Conflict of interest

The authors declare no conflict of interest.

### Ethical approval

This study was approved by the Organizational Ethics Committee of the School of Nursing, Midwifery, and Rehabilitation of Tehran University of Medical Sciences (Code: 95.01.08.1168).

### Consent to participate

Written informed consent was obtained from the participants before participation in the study. All the interviews were recorded with the permission of the participating parents using a voice recorder. The interview files were saved with a password as aliases under complete security.

### Funding

This study has been financially supported by the Tehran University of Medical Sciences.

### Authorship

 All authors collaborated extensively in the various stages of this study and the presentation of the article. MM, RM and ME conceived and designed the experiment.MM, RM, ME, collected data and AM, NLB analyzed data. MM, RM, ME, NLB and AM drafted and revised the paper.
